# Efficacy and safety of acupuncture for trigeminal neuralgia: A protocol for systematic review and meta-analysis

**DOI:** 10.1097/MD.0000000000022589

**Published:** 2020-10-02

**Authors:** Qinyu Zhao, Guang He, Zhenyan Zhang, Zhanbiao Li

**Affiliations:** a College of Acupuncture and Tuina, Shandong University of Traditional Chinese Medicine, Shandong; b Acupuncture Department; c Rehabilitation Department; d Pain Department, Liaocheng People's Hospital, Liaocheng, Shandong, PR China.

**Keywords:** acupuncture, meta-analysis, systematic review, trigeminal neuralgia

## Abstract

**Background:**

Trigeminal neuralgia (TN) is a disease accompanied by severe facial pain, which seriously affects the daily life of patients. Acupuncture is widely used by Traditional Chinese Medicine doctors to treat various painful diseases. Acupuncture combined with the treatment of trigeminal neuralgia can increase the analgesic effect and reduce side effects. However, there is still a lack of more quality multi-center clinical controlled trials and comprehensive meta-analysis, and a lack of more comprehensive and stronger evidence-based medical evidence.

**Methods:**

The 2 reviewers used the same search strategy to search CNKI, PubMed, Web of Science, Cochrane Library, Scopus, EBSCO, and the search date is until July 19, 2020. Two people read the retrieved literatures independently, and then delete duplications. Then, use the “risk of bias” tool in Cochrane Handbook 5.2 to score. Only documents with a score greater than 5 can be included. Make a table of literature characteristics, extract baseline patient data, research methods and possible risks of bias in the literature, interventions in treatment and control groups, outcome evaluation indicators (BNI, VAS, ER and AE), and research funding support. Use Review Manager 5.3.5 for meta-analysis, use Stata 15 for regression analysis to find the source of heterogeneity, and then perform subgroup analysis to resolve the heterogeneity based on the corresponding source.

**Results:**

The analysis of BNI, VAS, ER and AE data can provide high-quality evidence for high-quality synthesis and/or descriptive analysis of the effectiveness and safety of acupuncture treatment of various causes of urinary retention.

**Conclusion:**

This study can provide more comprehensive and strong evidence to prove whether acupuncture is effective and safe in the treatment of TN patients.

**Registration:**

The research has been registered and approved on the PROSPERO website. The registration number is CRD42019119606.

## 1 Introduction

### 1.1 Background introduction

Trigeminal neuralgia (TN) is a long-term facial pain disorder caused by 1 or more trigeminal nerve branch lesions, mostly unilateral. TN is mainly divided into 2 types: typical trigeminal neuralgia and atypical trigeminal neuralgia.[^1^,^2^] The typical trigeminal neuralgia causes extreme, occasional, sudden burning or electric-like facial pain that can last from a few seconds to up to 2 minutes. The induction of these pains can be continuous, and some can even be induced continuously for several hours. The atypical trigeminal neuralgia usually presents persistent low-intensity pain symptoms. Two types of trigeminal neuralgia can occur in combination. Although TN is not a pathogenic disease, its severe pain symptoms still severely reduce the patients quality of life.^[3]^ The mechanism of TN occurrence is caused by nerve demyelinating lesions, mostly caused by direct compression of blood vessels. It is generally believed that the most common cause of typical trigeminal neuralgia is the compression or other morphological changes of the trigeminal nerve by the blood vessels in the cerebellar cistern,^[1]^ while atypical trigeminal neuralgia is associated with multiple sclerosis plaques affecting the trigeminal nerve root or cerebellar pontine space-occupying lesions.[^4^,^5^] A systematic review of the incidence of trigeminal neuralgia has found that the incidence is between 0.03% (95% confidence interval 0.01–0.08) and 0.3% (95% confidence interval 0.16–0.55), so trigeminal neuralgia is a relatively rare disease.^[6]^ TN patients are significantly more women than men, and the elderly are at greater risk.[^7^,^8^] The treatment of TN is mainly divided into 2 ways, 1 is medical treatment and the other is surgical treatment. Carbamazepine has always been the first-line drug for the treatment of TN. Other antidepressants, antiepileptics and opioids are also often used to treat neuropathic pain. However, according to reports from the European Federation of Neurological Societies (EFNS) and the International Association for the Study of Pain Neuropathic Pain Special Interest Group (NeuPSIG), these drugs are often not always effective during the treatment process, and there are many side effects and even the risk of addiction.[^9^,^10^,^11^] Generally, surgery can be used when pain relief is insufficient or serious side effects or drug resistance are caused by medical treatment.^[12]^ Microvascular decompression (MVD) is the most commonly used surgical method to treat TN.^[13]^ Recently, some non-invasive radiotherapy has also been reported to be used to treat TN.[^14^,^15^]

### 1.2 Intervention method function introduction

Acupuncture is mostly used by Traditional Chinese Medicine doctors to treat pain in the clinic. Its analgesic effect has been widely recognized by people around the world since James Reston's article reported.^[16]^ The analgesic effect of acupuncture is mainly related to substance P and β-endorphin. It has been confirmed in animal experiments and clinical trials that acupuncture can reduce substance P and increase the expression of β-endorphin[^17^,^18^] Beta-endorphin is a morphine-like substance secreted by the hypothalamus and pituitary gland. It is excessively released due to pain, exercise, anxiety and depression. It binds to opioid receptors and inhibits the release of γ-aminobutyric acid (GABA) to play an analgesic effect.^[19]^ Substance P belongs to the tachykinin family, synthesized in the brain and spinal cord, and released in the sensory nerve endings of the skin, muscles and joints. Substance P binds to the (neurokinin) NK1 receptor on the spinal dorsal horn neurons to enhance pain.^[20]^ The reaction of β-endorphin with opioid receptors in nerve endings leads to a decrease in the secretion of tachykinins, especially substance P.^[21]^ In Traditional Chinese Medicine theory, the occurrence of pain is attributed to “pain caused by meridian obstruction” and “pain caused by the deficiency of qi and blood in meridian”. Acupuncture can achieve an analgesic effect by stimulating the acupoints to clear and activate the channels and collaterals. Acupuncture can be used as an auxiliary method to reduce the time of carbamazepine medication, thereby reducing side effects and reducing the patients operation rate. The EFNS and NeuPSIG guidelines for the treatment of neuropathic pain do not list acupuncture as an adjuvant treatment method, and further summary and research are needed to provide stronger clinical evidence. A systematic review and meta-analysis of the effectiveness and safety of acupuncture in the treatment of trigeminal neuralgia can provide sufficient clinical evidence.

## 2 Methods

This study has completed the registration of PROSPERO and obtained the approval number: CRD42019119606. This study strictly complies with the requirements of Cochrane Handbook 5.2.

### 2.1 Inclusion/exclusion criteria

A research report on the outcome evaluation methods of TN found that the outcome evaluation methods of TN are not uniform.^[22]^ To ensure that this study has less heterogeneity, we will only investigate the main outcome measures. The subjects were patients with clinically confirmed TN; studies were randomized controlled clinical trials (RCTs); acupuncture was used in the treatment group, with unrestricted acupoints or manoeuvres, and the control group was treated with carbamazepine; if the full text is still not available by contacting the author, we will exclude it; the study must have the following outcome measures: primary endpoints are Barrow Neurology Institute Pain Intensity Scale (BNI) and Visual Analogue Scale (VAS); secondary endpoints are overall effectiveness rate (ER), and safety outcome was adverse events (AE).

### 2.2 Study selection

This article will collect published Chinese and English literature on the treatment of TN with acupuncture. The literature comes from the following databases: China National Knowledge Infrastructure (CNKI), PubMed, Web of Science, Cochrane Library, Scopus, EBSCO. Search available literature from the establishment of the database to July 19, 2020 to find appropriate randomized controlled trials of acupuncture for the treatment of TN. The Chinese retrieval terms included “Mian Tong” or “San Cha Shen Jing Tong” (which means “TN”), “Zhen Jiu”, and “Sui Ji”, and the English retrieval terms included “Trigeminal neuralgia”, “TN MeSH”, “acupuncture”, “clinical trial”, “RCT”, “random”, and “randomization”. Based on the characteristics of different databases, retrieval strategies including both subject words + free words and keywords + full text were applied. The retrieval strategy is illustrated by the example used for searching PubMed shown in bellow (Table 1).

**Table 1:**
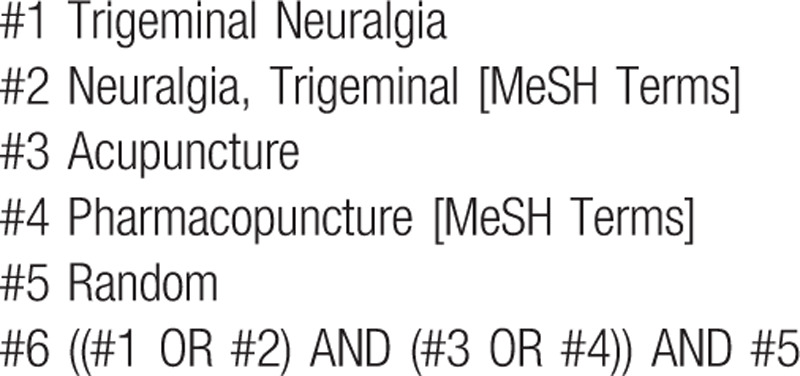
PubMed search strategy.

### 2.3 Study characteristics

All the steps of this study were carried out independently and simultaneously by 2 persons. If the 2 reviewers differed in their decision to include a study, the disagreement was resolved by discussion. If the consensus still cannot be achieved after discussing, we would seek a third party for advising (Zhanbiao Li). The reviewers searched the database and filtered all results independently (Qinyu Zhao and Guang He). The initially identified articles were imported into Note Express. According to the inclusion/exclusion criteria, and after reading the title and abstract of the literature, preliminary screening is performed and duplicate literature is removed. Full texts were acquired and read for eligible articles or articles that might meet the inclusion criteria to decide whether they would enter the final analysis. If there is a disagreement on the design of the publication or the results of the trial (for example, no safety issue is reported and no follow-up investigation is conducted), the corresponding author is contacted by phone or email to confirm the data we extracted from his publication.

### 2.4 Risk of bias within studies

Two reviewers used the Cochrane Handbook for Systematic Reviews of Interventions 5.2 to individually assess the risk of bias in the included literature, including the following: random sequence generation, allocation concealment, blinding of participants and personnel, blinding of outcome assessment, incomplete outcome data, selective reporting and other sources of bias. Among them, “other sources of bias” included the following:

1. Whether the experimental design is reasonable and feasible;
2. Whether the patient weight data is comparable;
3. Whether there is an obvious conflict of interest that leads to increased bias;
4. Whether the inclusion and exclusion criteria are clear.

Since it is difficult to achieve double-blind operation in acupuncture, in the “blinding of participants and personnel” item, as long as the single-blind principle is met, the risk of bias is defined as low risk. In terms of “selective reporting”, due to the lack of registration agreements in the included literature, if there are clear efficacy evaluation indicators, the risk of bias is defined as low. Each item is evaluated as low risk, and the corresponding literature gets 1 point. After the 2 reviewers separately assigned points, the results were compared. If there is a difference between the 2 reviewers in determining the document score, the difference can be resolved through discussion. If a consensus is still not reached after the discussion, we will seek third-party suggestions (Zhanbiao Li).

### 2.5 Data extraction

We have designed a literature feature table, which mainly includes the following aspects:

1. Baseline data of patients;
2. Research methods and possible risks of bias;
3. Interventions in treatment and control groups;
4. Outcome evaluation indicators, including BNI, VAS and ER;
5. Research fund support;
6. Adverse events.

The data were independently extracted and verified by 2 reviewers.

### 2.6 Statistical analysis

Quantitative analysis was performed by meta-analysis using Cochrane collaboration software Review Manager 5.3.5. Select relative risk (RR) for dichotomous data, and select mean difference (MD) and 95% confidence interval (CI) for continuous variables for statistical analysis. First, the Chi-Squared test and the heterogeneity test (*I* ^2^) are used for detection, and different data processing strategies are used according to different results. A fixed-effect model was used when *I* ^2^ was ≤50% and the *P* value was ≥.10; when *I* ^2^ was >50% or the *P* value was <.10, the random-effect model was applied. If the heterogeneity is high, we use Stata 15 for regression analysis to find the source of heterogeneity and then try to use subgroup analysis to increase the stability of the meta-analysis.

### 2.7 Measurements of publication bias

When the same endpoint contains a sufficient number of articles (more than 10 articles) to solve the same problem (more than 10 articles), the funnel chart is used to measure the publication bias.

### 2.8 Paper writing

**Figure 1:**
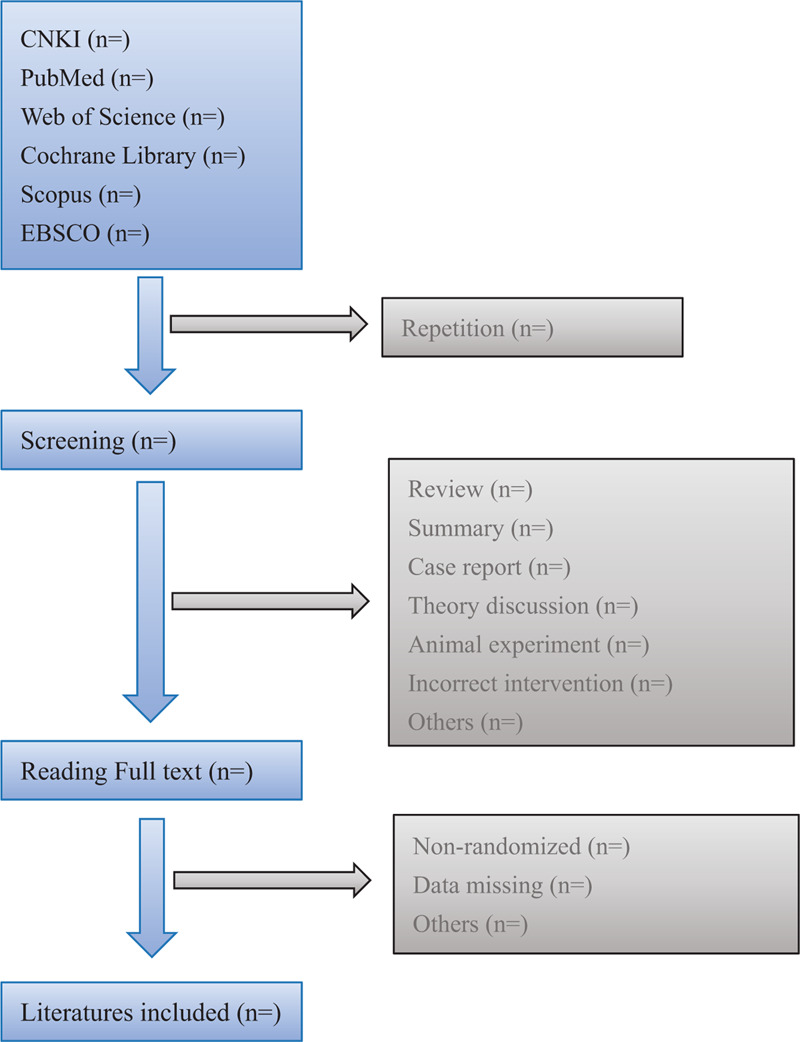
Flow diagram of literature retrieval.

During the writing of the thesis, the requirements of the PRISMA 2009 checklist should be strictly followed, and the research process should strictly follow the standard PRISMA 2009 flow chart (Fig. 1).

## 3 Discussion

TN is a severely painful disease that seriously affects the quality of life of patients. The main drugs used to treat trigeminal neuralgia are antiepileptic, antidepressant and opioid analgesics. These drugs often have side effects such as dizziness, dry mouth, and digestive symptoms, and some are also highly addictive. In the clinic of Traditional Chinese Medicine doctors, acupuncture has long been used to treat painful diseases, and the analgesic effect is often immediate, and it has the characteristics of simple operation, low side effects, and low treatment costs. Acupuncture combined with the treatment of TN can reduce the pain of patients and shorten the treatment period. However, there is still a lack of quality multi-center clinical controlled trials and comprehensive meta-analysis. This research will be conducted when there are enough high-quality literatures. To provide stronger evidence and better guide clinical practice, this study will be conducted in strict accordance with the requirements of Cochrane Handbook 5.2.

## Author contributions

**Conceptualization:** Qinyu Zhao.

**Data curation:** Qinyu Zhao, Guang He.

**Formal analysis:** Qinyu Zhao.

**Funding acquisition:** Zhanbiao Li.

**Methodology:** Qinyu Zhao, Zhanbiao Li.

**Software:** Qinyu Zhao, Guang He, Zhenyan Zhang.

**Supervision:** Zhenyan Zhang, Zhanbiao Li.

**Writing – original draft:** Qinyu Zhao.

**Writing – review & editing:** Qinyu Zhao.

## References

[1] Maarbjerg S Di Stefano G Bendtsen L Trigeminal neuralgia – diagnosis and treatment. Cephalalgia 2017;37:648–57. 28076964 10.1177/0333102416687280

[2] Cruccu G Finnerup NB Jensen TS Trigeminal neuralgia: new classification and diagnostic grading for practice and research. Neurology 2016;87:220–8. 27306631 10.1212/WNL.0000000000002840 PMC4940067

[3] Tolle T Dukes E Sadosky A Patient burden of trigeminal neuralgia: results from a cross-sectional survey of health state impairment and treatment patterns in six european countries. Pain Pract 2006;6:153–60. 17147591 10.1111/j.1533-2500.2006.00079.x

[4] Nomura T Ikezaki K Matsushima T Trigeminal neuralgia: differentiation between intracranial mass lesions and ordinary vascular compression as causative lesions. Neurosurg Rev 1994;17:51–7. 8078609 10.1007/BF00309988

[5] Jensen TS Rasmussen P Reske-Nielsen E Association of trigeminal neuralgia with multiple sclerosis: clinical and pathological features. Acta Neurol Scand 1982;65:182–9. 7080803 10.1111/j.1600-0404.1982.tb03076.x

[6] De Toledo IP Conti Réus J Fernandes M Prevalence of trigeminal neuralgia: a systematic review. J Am Dental Association 2016;147:570–6. 10.1016/j.adaj.2016.02.014 27017183

[7] Maarbjerg S Gozalov A Olesen J Trigeminal neuralgia - a prospective systematic study of clinical characteristics in 158 patients. J Head Face Pain 2014;54:1574–82. 10.1111/head.12441 25231219

[8] Katusic S Beard CM Bergstralth E Incidence and clinical features of trigeminal neuralgia, Rochester, Minnesota, 1945–1984. Ann Neurol 1990;27:89–95. 2301931 10.1002/ana.410270114

[9] Attal N Cruccu G Baron R EFNS guidelines on the pharmacological treatment of neuropathic pain: 2010 revision. Eur J Neurol 2010;17:1113–88. 20402746 10.1111/j.1468-1331.2010.02999.x

[10] Haanpää M Attal N Backonja M NeuPSIG guidelines on neuropathic pain assessment. Pain 2011;152:14–27. 20851519 10.1016/j.pain.2010.07.031

[11] Dworkin RH O Connor AB Kent J Interventional management of neuropathic pain: NeuPSIG recommendations. Pain 2013;154:2249–61. 23748119 10.1016/j.pain.2013.06.004 PMC4484720

[12] Green TH Girgis F Trigeminal neuralgia: medical management and surgical options. J Pain Palliative Care Pharmacotherapy 2019;33:32–3. 10.1080/15360288.2019.1624676 31369323

[13] Wang DD Ouyang D Englot DJ Trends in surgical treatment for trigeminal neuralgia in the United States of America from 1988 to 2008. J Clin Neurosci 2013;20:1538–45. 23932422 10.1016/j.jocn.2012.12.026

[14] Tavakol S Jackanich A Strickland BA Effectiveness of gamma knife radiosurgery in the treatment of refractory trigeminal neuralgia: a case series. Oper Neurosurg 2020;18:571–6. 10.1093/ons/opz311 31620790

[15] Tuleasca C Régis J Sahgal A Stereotactic radiosurgery for trigeminal neuralgia: a systematic review. J Neurosurg 2019;130:733–57. 10.3171/2017.9.JNS17545 29701555

[16] Reston J Now, let me tell you about my appendectomy in Peking. New York Times 1971;1.

[17] Mohammed N Allam H Elghoroury E Evaluation of serum beta-endorphin and substance P in knee osteoarthritis patients treated by laser acupuncture. J Complement and Inte Med 2018;15: doi: 10.1515/jcim-2017-0010. 10.1515/jcim-2017-0010 29303777

[18] Lee H Lee J Lee E Substance P and Beta endorphin mediate electroacupuncture induced analgesic activity in mouse cancer pain model. Acupuncture Electro 2009;34:27–40.

[19] Suh H Kim Y Choi Y Effects of GABA receptor antagonists injected spinally on antinociception induced by opioids administered supraspinally in mice. Eur J Pharmacol 1996;307:141–7. 8832215 10.1016/0014-2999(96)00226-9

[20] Herbert MK Holzer P Warum versagen Substanz P (NK1)-Rezeptorantagonisten in der Schmerztherapie? Der Anaesthesist 2002;51:308–19. 12063723 10.1007/s00101-002-0296-7

[21] Sweet WH Neuropeptides and monoaminergic neurotransmitters: their relation to pain. J Roy Soc Med 2018;73:482–91. 10.1177/014107688007300704 PMC1437682 6164791

[22] Nova CV Zakrzewska JM Baker SR Treatment outcomes in trigeminal neuralgia–a systematic review of domains, dimensions and measures. World Neurosurg 2020;6:100070. 10.1016/j.wnsx.2020.100070 PMC7036566 32123867

